# A Comparative Analysis of Intraoperative Assessment Versus Preoperative Computed Tomography in Determining Aditus Ad Antrum Patency in Mucosal Chronic Otitis Media

**DOI:** 10.3390/medicina62071359

**Published:** 2026-07-14

**Authors:** Goran Latif Omer, Sahand Soran Ali, Giuseppe De Donato, Andrea Gravina, Stefano Berrettini, Luca Bruschini, Stefano Di Girolamo

**Affiliations:** 1 Department of Clinical Sciences, University of Sulaimani, Sulaymaniyah 46001, Iraq; 2 Department of Otorhinolaryngology–Head and Neck Surgery, Tor Vergata University of Rome, 00133 Rome, Italy; gdedonato2@gmail.com (G.D.D.); andreagravina1993@gmail.com (A.G.); stefano.di.girolamo@uniroma2.it (S.D.G.); 3 College of Pharmacy, American University of Iraq, Sulaymaniyah 46001, Iraq; sahand.soran@auis.edu.krd; 4 Department of Surgical, Medical, Molecular and Critical Area Pathology, University of Pisa, 56126 Pisa, Italy; stefano.berretti@unipi.it (S.B.); luca.bruschini@unipi.it (L.B.)

**Keywords:** otitis media, tomography, X-ray computed, mastoid, tympanoplasty, mastoidectomy, ear, middle

## Abstract

*Background and Objectives*: Patency of the aditus ad antrum is essential for middle ear and mastoid ventilation and may influence surgical outcomes in chronic otitis media (COM). Although preoperative computed tomography (CT) is commonly used to assess patency, its reliability remains uncertain. This study compared preoperative CT findings with intraoperative saline and suction testing and evaluated the impact of intraoperative assessment on surgical planning. *Materials and Methods*: This observational study included 55 patients with mucosal COM who underwent middle ear surgery with preoperative CT assessment of the aditus. After limited antrostomy, aditus patency was assessed intraoperatively using saline and suction tests, which served as the reference standard. CT and intraoperative findings were cross-tabulated, diagnostic accuracy for CT-detected obstruction was calculated, and changes in surgical planning were recorded. Associations were tested using the chi-square test, with significance set at *p* < 0.05. Analysis was performed using SPSS version 22.0. *Results*: Intraoperative testing showed a patent aditus in 31 patients (56.4%), while CT indicated patency in 19 (34.5%). CT and intraoperative findings were concordant in 23 cases (41.8%) and discordant in 32 (58.2%), with a Cohen kappa of −0.12. CT detected obstruction with 58.3% sensitivity, 29.0% specificity, 38.9% positive predictive value, 47.4% negative predictive value, and 41.8% accuracy. Intraoperative findings changed the surgical plan in 26 patients (47.3%). Intraoperative patency was significantly associated with antral mucosal appearance (*p* < 0.001). *Conclusions*: Preoperative CT alone may not reliably assess aditus ad antrum patency. Intraoperative saline and suction testing provides a simple, practical method to confirm patency and guide mastoid surgery.

## 1. Introduction

Ventilation of the middle ear cleft maintains pressure balance and gas exchange [1]. A key conduit within this cleft is the aditus ad antrum, the passage between the mastoid antrum and the epitympanum (attic) [2]. Antrostomy opens the mastoid antrum and allows assessment of mastoid air-cell aeration, and it is frequently performed during mastoid surgery and as part of myringoplasty [3,4]. Acute mastoiditis, chronic mastoiditis, and the complications of chronic otitis media are indications for mastoid surgery, the overall aim of which is a safe and dry ear [5,6]. Mastoidectomy is broadly classified as canal-wall-up or canal-wall-down. In canal-wall-up surgery the posterior bony external auditory canal is preserved; canal-wall-down surgery is reserved for extensive chronic disease and recurrent cholesteatoma [4].

Preoperatively, surgeons may order temporal bone computed tomography (CT) to evaluate anatomy and plan surgery [7]. CT of the aditus ad antrum can also be used to judge its patency, which some authors regard as having prognostic value in chronic otitis media (COM) [8]. However, CT is not indicated in every case. Patency can also be checked intraoperatively by the saline and suction tests. For the suction test, a piece of cotton placed in the external auditory canal is soaked with normal saline; suction is then applied at the antrostomy, and patency is confirmed when the cotton is drawn through the aditus and dries, or when movement of the cotton is seen. For the saline test, patency is confirmed when saline flushed into the mastoid cavity flows through the aditus into the middle ear and out of the external auditory canal [9]. These tests confirm adequate middle ear ventilation, which, if not addressed, can compromise the postoperative outcome [10].

Although preoperative CT has been described as a reliable means of assessing aditus patency and planning surgery [11], the present authors contend that CT should not be relied on in isolation. CT is not always indicated, and discordance can occur between imaging and intraoperative findings: a falsely negative CT may lead to an obstructed aditus being overlooked, whereas apparent obstruction on CT may prompt unnecessary radical surgery in an ear with a patent aditus. This study therefore assessed aditus patency intraoperatively through a limited antrostomy and examined its agreement with preoperative CT, in order to quantify the discrepancy between the two methods and the diagnostic accuracy of CT for detecting an obstructed aditus ad antrum.

## 2. Materials and Methods

### 2.1. Study Design and Patient Population

This observational study was conducted on patients with mucosal COM who underwent middle ear surgery and had a preoperative CT of the aditus. CT had been obtained either because of ear discharge or to evaluate suspected anatomical variation. The study ran from 1 November 2024 to 1 May 2025. Fifty-five patients were included, none of whom had cholesteatoma. Patients were followed at 2, 4, 6, and 8 weeks postoperatively.

### 2.2. Eligibility Criteria

Patients with mucosal COM (a pars tensa or central perforation) undergoing primary surgery who had a preoperative CT obtained for discharge or suspected anatomical variation were eligible. Patients with cholesteatoma, attic pathology, or visible middle ear granulation tissue, and those undergoing revision surgery, were excluded because they required a different type of mastoidectomy.

### 2.3. Computed Tomography Acquisition and Interpretation

**Figure 1:**
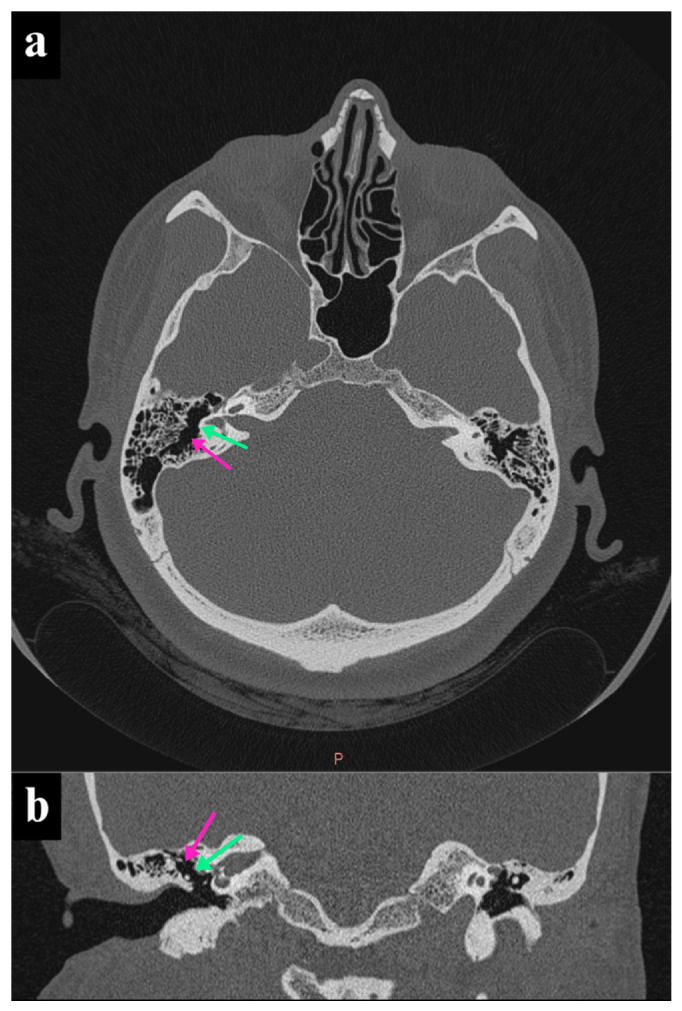
High-resolution CT of the right temporal bone showing a patent aditus ad antrum. (**a**) The axial image shows a patent aditus ad antrum (green arrow) and a normal, non-opacified antrum (purple arrow). (**b**) The coronal image again shows a patent aditus ad antrum (green arrow) and a normal, non-opacified antrum (purple arrow).

**Figure 2:**
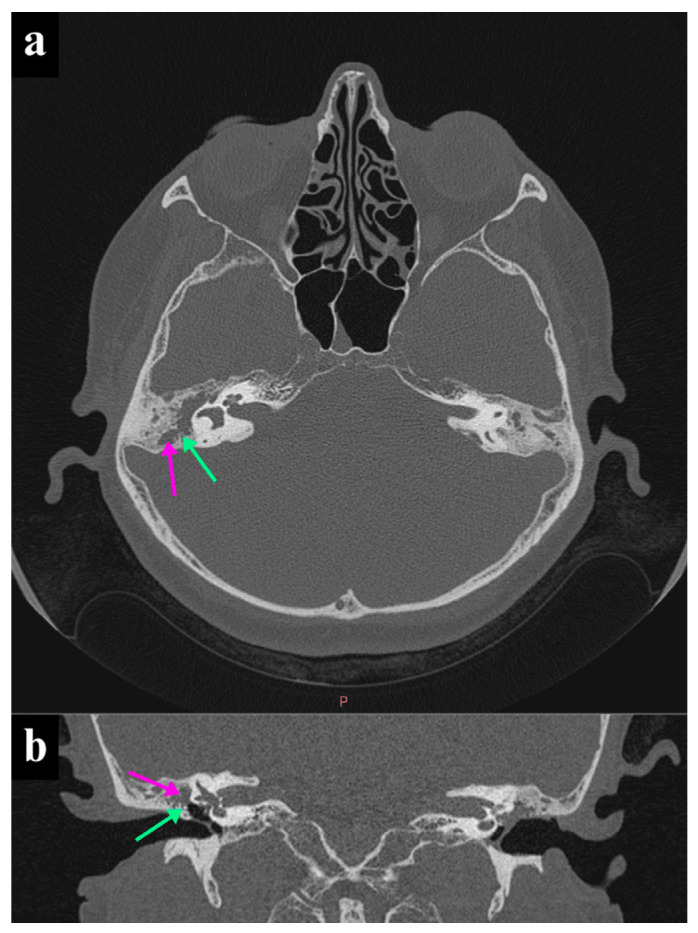
High-resolution CT of the right temporal bone showing an obstructed aditus ad antrum. (**a**) The axial image shows an obstructed aditus ad antrum (green arrow) and opacification of the antrum (purple arrow). (**b**) The coronal image again shows an obstructed aditus ad antrum (green arrow) and opacification of the antrum (purple arrow).

High-resolution CT of the temporal bone was performed using a 64-slice multidetector CT scanner (SOMATOM, Siemens Healthineers, Erlangen, Germany). Images were acquired without intravenous contrast using thin-section axial acquisition through the petrous temporal bones, with subsequent multiplanar reconstruction in the axial and coronal planes. Typical scan parameters were 120 kVp, automatic tube-current modulation (approximately 100–250 mAs), a slice thickness of 0.5–0.625 mm, a slice interval of 0.3–0.6 mm, a pitch of approximately 0.5–1.0, and a small field of view focused on the temporal bones. Images were reconstructed with a high-spatial-frequency bone algorithm and reviewed on a bone window setting. The aditus ad antrum was classified as patent when a continuous air column was visible between the epitympanum and the mastoid antrum on contiguous sections, and as obstructed when this air column was interrupted by soft-tissue density or by sclerosis. All preoperative CT images were interpreted prospectively, before surgery, by a radiologist who was blinded to the intraoperative findings, and the radiological assessment was recorded before the intraoperative test was performed. In patients with bilateral disease, only the ear that underwent surgery during the study period was included, and a single observation per patient was analyzed (Figure 1 and Figure 2).

### 2.4. Procedure

**Figure 3:**
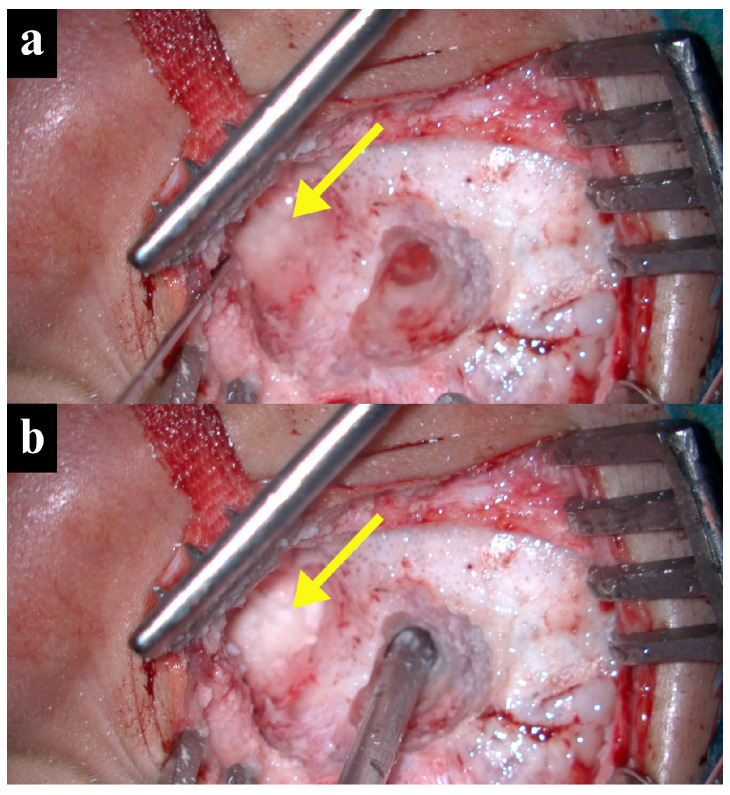
Left-sided antrostomy showing a patent aditus ad antrum during the early steps of tympanoplasty. The antrostomy appears as the opening in the mastoid; the cotton is placed in the external auditory canal (arrow). In (**a**) the cotton is soaked with normal saline; in (**b**), application of suction at the antrostomy draws the saline from the cotton, which dries, indicating a patent aditus ad antrum.

**Figure 4:**
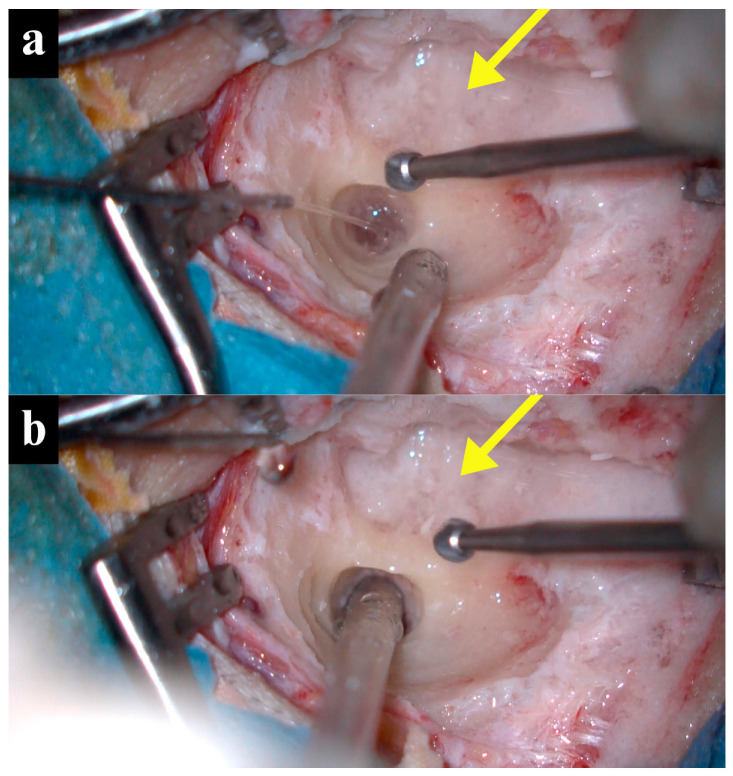
Right-sided antrostomy showing an obstructed aditus ad antrum. In (**a**), cotton soaked with normal saline is placed in the external auditory canal (arrow) and a suction device is positioned at the antrostomy. In (**b**), application of suction does not dry the cotton, indicating an obstructed aditus ad antrum and absence of adequate ventilation.

Eligible patients first had aditus patency assessed on the preoperative CT and were evaluated for fitness for anesthesia and surgery. Intraoperatively, immediately after a limited antrostomy, patency was assessed by both the suction and saline tests. For the suction test, a piece of cotton placed in the external auditory canal was soaked with normal saline; suction was then applied at the antrostomy, and patency was confirmed when the cotton was drawn through the aditus and dried, or when movement of the cotton was observed. For the saline test, patency was confirmed when saline flushed into the mastoid cavity flowed through the aditus into the middle ear and out of the external auditory canal. The intraoperative test served as the reference standard against which CT was compared (Figure 3 and Figure 4).

### 2.5. Statistical Analysis

Sociodemographic and clinical variables were summarized as frequencies and percentages; age was expressed as mean ± standard deviation. The chi-square test was used to examine associations between categorical variables. Agreement between CT and the intraoperative test was assessed using the proportion of concordant cases and the Cohen kappa coefficient. The diagnostic accuracy of CT for detecting an obstructed aditus was expressed as sensitivity, specificity, positive and negative predictive values, and overall accuracy, using the intraoperative test as the reference standard. The level of statistical significance was set at *p* < 0.05. Analysis was performed with SPSS version 22.0 (IBM Corp., Armonk, NY, USA).

### 2.6. Ethics Approval

This study was approved by the Ethics Committee of the College of Medicine, University of Sulaimani (approval number: 62, date: 4 August 2024). All procedures were in accordance with the Declaration of Helsinki, and informed consent was obtained from all participants.

## 3. Results

Fifty-five patients with a mean age of 36.6 ± 10.8 years were studied. Both intraoperative tests yielded identical results in every patient, and all ears were of the mucosal (safe) type. No patient reported tinnitus as a presenting complaint.

Sociodemographic and clinical findings are shown in Table 1. The 31–40-year group was most common (19 patients, 34.5%), and the sexes were almost equal (27 men, 49.1%; 28 women, 50.9%). The commonest complaint was ear discharge (32 patients, 58.2%), followed by hearing impairment (12 patients, 21.8%). Disease was right-sided in 20 patients (36.4%), left-sided in 21 (38.2%), and bilateral in 14 (25.5%). A central perforation was most frequent (23 patients, 41.8%), followed by anterior (15 patients, 27.3%) and subtotal perforations (17 patients, 30.9%). Tympanosclerosis was present in 19 patients (34.5%).

Investigation findings are summarized in Table 2. The intraoperative saline/suction test showed a patent aditus in 31 patients (56.4%) and an obstructed aditus in 24 (43.6%). Preoperative CT indicated a patent aditus in only 19 patients (34.5%) and an obstructed aditus in 36 (65.5%). The intraoperative test altered the CT-based surgical plan in 26 patients (47.3%). The commonest radiological finding was partial-to-complete sclerosis of the mastoid air cells (18 patients, 32.7%), followed by stagnant fluid limited to the posterior epitympanum (9 patients, 16.4%). On clinical inspection of the antrum, 31 patients (56.4%) had healthy mucosa, 10 (18.2%) slight inflammation, 10 (18.2%) moderate inflammation, and 4 (7.3%) severe inflammation with granulation tissue.

Associations of the radiological findings with the CT-based assessment, the impact on the surgical plan, the perforation site, and tympanosclerosis are shown in Table 3. Of the 12 patients with a normal CT, 11 (91.7%) had a patent aditus on CT. Of the 17 patients with stagnant fluid in the epitympanum (whole or posterior), 15 (88.2%) did not have their surgical plan altered, whereas among the 12 patients with a normal CT, 9 (75.0%) had their plan altered by the intraoperative test. A central perforation was present in 8 (66.7%) of the 12 patients with a normal CT, and 12 (66.7%) of the 18 patients with mastoid sclerosis had tympanosclerosis.

The relationship of the intraoperative test with the change in surgical plan and with the antral mucosal appearance is shown in Table 4. Of the 26 patients whose surgical plan was altered, 11 (42.3%) had a patent aditus and 15 (57.7%) an obstructed aditus on intraoperative testing (*p* = 0.047). A highly significant association was found with the antral mucosa (*p* < 0.001): all 31 ears with healthy mucosa had a patent aditus, whereas all 24 ears with inflammation, regardless of severity, had an obstructed aditus.

The cross-tabulation of preoperative CT against the intraoperative test is shown in Table 5. CT and the intraoperative test agreed in 23 patients (41.8%) and disagreed in 32 (58.2%); the Cohen kappa was −0.12, indicating agreement no better than chance. Among the 36 patients with an obstructed aditus on CT, 22 (61.1%) were found to have a patent aditus intraoperatively; among the 19 patients with a patent aditus on CT, 10 (52.6%) were obstructed intraoperatively. Using the intraoperative test as the reference standard, CT detected an obstructed aditus with a sensitivity of 58.3% (14/24), a specificity of 29.0% (9/31), a positive predictive value of 38.9% (14/36), a negative predictive value of 47.4% (9/19), and an overall accuracy of 41.8% (23/55).

## 4. Discussion

Chronic suppurative otitis media (CSOM) can cause hearing loss, otorrhea, and tympanic membrane perforation; when the membrane is perforated, tympanoplasty is required to restore drainage and ventilation of the tympanic cavity [12]. Besides eustachian tube function, the aditus ad antrum is crucial for ventilation of the mastoid air cells, and failure of aeration during tympanoplasty is an important cause of surgical failure in mucosal COM [11]. Patency can be assessed preoperatively by CT or intraoperatively by the saline or suction test. In a study confined to mucosal COM with sclerosed mastoid, Varma et al. reported complete concordance between HRCT and intraoperative findings: all 23 ears patent on HRCT were patent intraoperatively, and all 12 ears blocked on HRCT were blocked intraoperatively (*p* < 0.001), suggesting perfect agreement [11]. In contrast, our unselected mucosal COM population showed marked discordance: of the 31 patients with an intraoperatively patent aditus, 22 (71.0%) had an obstructed aditus on CT, and of the 24 with an intraoperatively obstructed aditus, 14 had an obstructed aditus on CT. With a sensitivity of only 58.3%, a specificity of only 29.0%, and a Cohen kappa of −0.12, our data underscore the need for intraoperative confirmation of patency. The restriction of the study by Varma et al. to sclerosed mastoids, in which radiological obstruction may correspond more closely to true obstruction, may partly account for this difference.

Despite this difference in agreement, the prevalence of a patent aditus was broadly similar across studies. In the study by Varma et al., 23 of 35 patients (65.7%) had a patent aditus, comparable to our 56.4% [11]. A similar prevalence was reported by Lakhawat et al., in whom 59 of 100 patients (59%) had a patent aditus [9]. A higher patency of 80% was reported in the 50-patient study by Bassiouny et al. [13].

This study also found a highly significant association (*p* < 0.001) between the intraoperative test and the clinical appearance of the antral mucosa: all ears with healthy mucosa had a patent aditus, whereas all ears with inflammation, regardless of severity, had an obstructed aditus. Intraoperative testing can guide whether the CT-based plan should be modified, either toward more extensive surgery when unexpected obstruction is found, or away from it when the aditus proves patent despite apparent obstruction on CT, thereby sparing healthy antral and air-cell mucosa. The surgical plan was altered in 26 of the 55 patients (47.3%).

Among the 26 patients whose management was modified after the intraoperative test, the direction of the change corresponded to the intraoperative patency finding and the appearance of the antral mucosa. In the 15 patients in whom the aditus was found to be obstructed, all of whom had inflamed antral mucosa (slight in 5, moderate in 8, and severe in 2), management was extended beyond the initially planned tympanoplasty to address ventilation, comprising wider opening of the aditus and clearance of obstructing mucosal or sclerotic tissue, with more formal mastoid exploration where the obstruction could not be relieved through the limited antrostomy. In the remaining 11 patients, in whom the aditus was found to be patent and the antral mucosa healthy, surgery was confined to tympanoplasty and the more extensive mastoid work that might otherwise have been considered was not undertaken, preserving the healthy antral and air-cell mucosa. The decision to modify the plan therefore reflected the combined intraoperative assessment of patency and mucosal state rather than the preoperative CT appearance alone, consistent with the poor agreement between CT and the intraoperative findings reported above.

A limitation directly relevant to the interpretation of these changes is that the present study was designed to compare radiological and intraoperative assessment of patency, not to measure the downstream clinical benefit of acting on the intraoperative finding. Structured postoperative outcome measures, such as graft uptake, resolution of otorrhea, and the need for revision surgery, were not collected in a manner that would allow a valid comparison between patients whose surgical plan was altered and those whose plan was unchanged, and the follow-up period of eight weeks would in any case be too short to capture graft failure or recurrence reliably. We have therefore refrained from reporting such outcomes rather than present underpowered or incomplete data. A prospective study with a defined outcome set and longer follow-up, comparing altered and unaltered groups, is needed to confirm that intraoperative decision-making translates into improved surgical results; we regard this as the most important direction for future work.

This study has limitations. First, the relatively small sample limits generalizability. Second, the exclusion of patients with cholesteatoma and those undergoing revision surgery means the findings cannot be extrapolated to more complex disease. Third, both CT interpretation and the intraoperative tests are operator dependent, and the saline/suction tests, although practical, are not formally validated against an independent gold standard.

## 5. Conclusions

This study highlights the value of the saline/suction test in confirming aditus ad antrum patency. Only a limited proportion of patients undergoing middle ear surgery have an indication for preoperative CT, and even when CT is available its agreement with intraoperative findings was weak. Relying on imaging alone may therefore lead to overlooked obstruction or, conversely, unnecessary damage to mastoid and air-cell mucosa through extensive surgery when the aditus is in fact patent. Intraoperative confirmation with the simple saline and suction tests helps ensure accurate surgical planning, prevents overtreatment, and preserves healthy mastoid structures whenever possible.

## Figures and Tables

**Table 1:** Sociodemographic and clinical characteristics of the 55 patients.

Variable	Frequency, *n* (%)
Mean age, years (mean ± SD)	36.6 ± 10.8
Age group	
≤20 years	4 (7.3)
21–30 years	13 (23.6)
31–40 years	19 (34.5)
41–50 years	12 (21.8)
51–60 years	7 (12.7)
≥61 years	0 (0.0)
Sex	
Male	27 (49.1)
Female	28 (50.9)
Presenting complaint	
Hearing impairment	12 (21.8)
Ear discharge	32 (58.2)
Discharge and hearing impairment	11 (20.0)
Side of disease	
Right	20 (36.4)
Left	21 (38.2)
Bilateral	14 (25.5)
Site of perforation	
Central	23 (41.8)
Anterior	15 (27.3)
Posterior	0 (0.0)
Marginal	0 (0.0)
Subtotal	17 (30.9)
Tympanosclerosis	
Present	19 (34.5)
Absent	36 (65.5)
Mucosal status	
Safe (mucosal)	55 (100.0)
Unsafe (squamous)	0 (0.0)
Total	55 (100.0)

SD, standard deviation.

**Table 2:** Investigation findings.

Variable	Frequency, *n* (%)
Saline/suction test	
Patent aditus	31 (56.4)
Obstructed aditus	24 (43.6)
Preoperative CT of the aditus	
Patent aditus	19 (34.5)
Obstructed aditus	36 (65.5)
Saline/suction test altered the surgical plan	
Yes	26 (47.3)
No	29 (52.7)
Radiological findings	
Partial-to-complete sclerosis of mastoid air cells	18 (32.7)
Stagnant fluid in mastoid antrum	8 (14.5)
Stagnant fluid in whole epitympanum	8 (14.5)
Stagnant fluid in posterior epitympanum only	9 (16.4)
Signs of cholesteatoma	0 (0.0)
Normal	12 (21.8)
Clinical findings of mastoid antrum	
Healthy antral mucosa	31 (56.4)
Slight inflammation	10 (18.2)
Moderate inflammation	10 (18.2)
Severe inflammation with granulation tissue	4 (7.3)
Total	55 (100.0)

CT, Computed Tomography.

**Table 3:** Association of radiological findings with the CT-based aditus assessment, change in surgical plan, perforation site, and tympanosclerosis.

Variable		Sclerosis of Air Cells	Fluid in Antrum	Fluid in Whole Epitympanum	Fluid in Post. Epitympanum	Cholesteatoma	Normal	*p*
Preoperative CT of aditus	Patent	2 (11.1)	2 (25.0)	1 (12.5)	3 (33.3)	0 (0.0)	11 (91.7)	<0.001
	Obstructed	16 (88.9)	6 (75.0)	7 (87.5)	6 (66.7)	0 (0.0)	1 (8.3)	
Surgical plan altered	Yes	9 (50.0)	6 (75.0)	0 (0.0)	2 (22.2)	0 (0.0)	9 (75.0)	0.004
	No	9 (50.0)	2 (25.0)	8 (100.0)	7 (77.8)	0 (0.0)	3 (25.0)	
Perforation site	Central	9 (50.0)	1 (12.5)	2 (25.0)	3 (33.3)	0 (0.0)	8 (66.7)	<0.001
	Anterior	2 (11.1)	7 (87.5)	3 (37.5)	0 (0.0)	0 (0.0)	3 (25.0)	
	Subtotal	7 (38.9)	0 (0.0)	3 (37.5)	6 (66.7)	0 (0.0)	1 (8.3)	
Tympanosclerosis	Present	12 (66.7)	2 (25.0)	2 (25.0)	0 (0.0)	0 (0.0)	3 (25.0)	0.007
	Absent	6 (33.3)	6 (75.0)	6 (75.0)	9 (100.0)	0 (0.0)	9 (75.0)	

Values are *n* (%) within each radiological category column. CT, computed tomography. *p* values are from the chi-square test and are unadjusted for multiple comparisons; they should therefore be interpreted as exploratory.

**Table 4:** Relationship of the saline/suction test with the change in surgical plan and with the clinical appearance of the antral mucosa.

Variable		Patent Aditus	Obstructed Aditus	*p*
Surgical plan altered	Yes (*n* = 26)	11 (35.5)	15 (62.5)	*p* = 0.047
	No (*n* = 29)	20 (64.5)	9 (37.5)	
Clinical findings of antral mucosa	Healthy mucosa	31 (100.0)	0 (0.0)	*p* < 0.001
	Slight inflammation	0 (0.0)	10 (41.7)	
	Moderate inflammation	0 (0.0)	10 (41.7)	
	Severe inflammation	0 (0.0)	4 (16.7)	

Percentages in the table are column percentages (within each saline/suction test result); percentages quoted in the text for the altered-plan group are row percentages (of the 26 patients whose plan was altered). *p* values are from the chi-square test and are unadjusted for multiple comparisons and should be interpreted as exploratory.

**Table 5:** Cross-tabulation of preoperative CT against the intraoperative saline/suction test (reference standard).

Variable		Patent (*n* = 31)	Obstructed (*n* = 24)
Preoperative CT of aditus	Patent (*n* = 19)	9	10
	Obstructed (*n* = 36)	22	14

Values are numbers of patients. Treating the intraoperative saline/suction test as the reference standard and CT as a test for obstruction: sensitivity 58.3% (14/24), specificity 29.0% (9/31), positive predictive value 38.9% (14/36), negative predictive value 47.4% (9/19), and overall accuracy 41.8% (23/55). Agreement between the two methods: 41.8%; Cohen kappa = −0.12. CT, computed tomography.

## Data Availability

The data presented in this study are available on request from the corresponding author.

## Abbreviations

The following abbreviations are used in this manuscript:

COM	Chronic otitis media
CSOM	Chronic suppurative otitis media
CT	Computed tomography
HRCT	High-resolution computed tomography
